# Molecular pharmacology in a simple model system: Implicating MAP kinase and phosphoinositide signalling in bipolar disorder

**DOI:** 10.1016/j.semcdb.2010.11.002

**Published:** 2011-02

**Authors:** Marthe H.R. Ludtmann, Katrina Boeckeler, Robin S.B. Williams

**Affiliations:** Centre for Biomedical Sciences, School of Biological Sciences, Royal Holloway University of London, Egham TW20 0EX, UK

**Keywords:** DAG, diacylglycerol, GSK3/A, mammalian/*Dictyostelium* glycogen synthase kinase 3/A, IMPase, inositol monophophatase, IPPase, Inositol polyphosphate phosphatase, InsP_3_, inositol 1,4,5-triphosphate, 2M2P, 2-methyl-2-pentenoic acid, PI, phosphatidylinositol, PIP_2_, phosphatidylinositol 4,5-biphosphate, PI3K, phosphatidylinositol 3-kinase, PIP, phosphatidylinositol monophosphate, PO, prolyl oligopeptidase, MAPK, mitogen activated protein kinase, MEK, MAPK kinase, MEKK, MEK kinase, MKP, MAP kinase phosphatase, PKA, protein kinase A, PLC, phospholipase C, REMI, restriction enzyme mediated integration, VPA, valproic acid, Bipolar disorder, *Dictyostelium*, Lithium, MAP kinase, Pharmacology, Phosphoinositol, Valproic acid

## Abstract

Understanding the mechanisms of drug action has been the primary focus for pharmacological researchers, traditionally using rodent models. However, non-sentient model systems are now increasingly being used as an alternative approach to better understand drug action or targets. One of these model systems, the social amoeba *Dictyostelium*, enables the rapid ablation or over-expression of genes, and the subsequent use of isogenic cell culture for the analysis of cell signalling pathways in pharmacological research. The model also supports an increasingly important ethical view of research, involving the reduction, replacement and refinement of animals in biomedical research. This review outlines the use of *Dictyostelium* in understanding the pharmacological action of two commonly used bipolar disorder treatments (valproic acid and lithium). Both of these compounds regulate mitogen activated protein (MAP) kinase and inositol phospholipid-based signalling by unknown means. Analysis of the molecular pathways targeted by these drugs in *Dictyostelium* and translation of discoveries to animal systems has helped to further understand the molecular mechanisms of these bipolar disorder treatments.

## 1 Introduction

Bipolar disorder, a neurological condition that causes cyclic variation in mood, gives rise to a devastating effect on quality of life and significantly increases the chance of suicide [1]. To improve diagnosis and treatment of bipolar disorder, ongoing research has focused on identifying the molecular basis of the condition. Research models used for this purpose have traditionally been rodents. However, the increasing use of other simple model systems such as the social amoeba, *Dictyostelium discoideum*, provides distinct advantages. The following review will introduce bipolar disorder and *Dictyostelium* as a biomedical model. The review will then focus on two of the most widely used bipolar disorder treatments – valproic acid (VPA) and lithium – by firstly outlining current knowledge of cell signalling pathways regulated by each treatment, and then illustrating the use of *Dictyostelium* (and subsequent translation to mammalian systems) to enhance our understanding of putative bipolar disorder-dependent cell signalling changes in both pathways.

## 2 Bipolar disorder background

Bipolar disorder (also known as manic depression) is a neurological condition giving rise to cyclic and extreme changes in mood. The two most widely occurring types of bipolar disorder are characterised by slightly different re-occurring states: type 1 describes recurrent mood swings from depression to mania, whilst type 2 describes recurrent mood swings from depression to euthymic (stabilised mood) behaviour or mild forms of mania [2–4]. Bipolar disorder is amongst the most common serious neurological disorders, with estimates of its worldwide occurrence of up to 4% [4–6]. An equal number of men and women develop this illness, and its frequency is highest in people from a low economic bracket or in offspring of those in higher socio-economic indices [7]. In the UK the financial burden on the health system amounts to £4.6 billion annually [8] and although it does not in itself cause physical bodily damage, it gives rise to emotionally damaging behaviour and bears a 15% risk of suicide when left untreated [9]. A recent US study suggests that bipolar disorder occurrence doubled in adults and increased by 40-fold for those under 20 year olds from 1994 to 2003 [10]. Many studies suggest an heritable risk, since monozygotic twins indicate a 60% co-inheritance which decreases to 7% for first-degree relatives [11].

Current treatments for bipolar disorder include lithium, anticonvulsant drugs (carbamazepine, Tegretol), valproic acid (Depakote), gabapentin (Neurontin) and lamotrigine (Lamictal), antidepressants such as bupropion (Wellbutrin) or sertraline (Zoloft), neuroleptics (e.g. haloperidol) and benzodiazepines (e.g. lorazepam). These treatments require continuous long-term use and are thus non-curative. A decision on which treatment to use is normally based on individual symptoms. Poor response rates to drug treatment (only marginally better than placebo) occur in approximately 35–50% of patients, with significant side effects that often lead to non-adherence [12].

Research into bipolar disorder remains a hugely complex undertaking. This is primarily since there are no well accepted models for the condition [13], patients cannot be taken off treatment since this may give rise to damaging mood changes, and since the molecular mechanism of bipolar disorder drugs have not been discovered. For these reasons current treatments have been found as a secondary effect whilst treating other conditions (e.g. epilepsy). However, three broad approaches continue to be used in bipolar disorder research: genetics, physiology and pharmacology.

• *Genetics:* Identifying the genetic basis of bipolar disorder is likely to provide the ultimate origin of the condition and is thus a fundamentally important approach. There is clear evidence that the occurrence of bipolar disorder has a strong genetic component [14] and a range of specific loci have been associated with the disorder [15,16]. However, as a consequence of the large number of loci involved, this approach is highly complex.
• *Biochemistry:* Understanding physical changes in individuals with bipolar disorder may provide an avenue to identify the molecular basis of the condition. For example, recent studies have shown bipolar disorder-dependent changes in brain volume [17,18] brain imaging studies [19], in gene expression [20], enzymatic activity [21] and in the regulation of cellular components such as fatty acids [22,23].
• *Pharmacology:* The identification and characterisation of changes in cell signalling caused by bipolar disorder treatments may both identify the molecular mechanism causing bipolar disorder, and enable the development of more efficacious treatments or treatments with reduced side effects. The vast array of genetic risk factors associated with bipolar disorder occurrence is expected to give rise to changes in only a limited number of signalling pathways that are then controlled by a small number of current bipolar disorder treatments. Thus understanding bipolar disorder-associated signalling pathways may help the treatment of patients with a wide array of causative factors. However, this approach is also complex since bipolar disorder treatments have numerous effects and/or targets, and the primary sites of action are often unknown. The majority of research into the molecular basis of bipolar disorder treatments has centred on valproic acid (VPA) and lithium.

## 3 *Dictyostelium* as a model for pharmacology

The use of invertebrate, non-sentient model organisms can facilitate our understanding of the mechanism of drug action on cellular level whilst reducing the use of animals in research. *Dictyostelium discoideum* is one of these models listed as one of ten non-mammalian biomedical models recognised by the US National Institutes of Health (NIH). *Dictyostelium* is more complex than other models listed such as *Sacchromycies cerevisiae* (baker's yeast), *Schizosaccharomyces pombe* (fission yeast) *or Neurospora crassa* (filamentous fungus) since *Dictyostelium* cells undergo chemotactic and random movement. It also has rudimentary development mechanisms and related signalling pathways. On the other hand, *Dictyostelium* is simpler than other models listed such as *Caenorhabditis elegans* (round worm) and *Drosophila melanogaster* (fruit fly), *Danio rerio* (zebrafish), and *Xenopus tropicalis* (frog) since it lacks the much more complex developmental processes involved in the formation of these animals and lacks their basic neurological networks. *Dictyostelium* has evolved a strong niche for studies into cell movement and development [24–26], for understanding the cellular role of a range of proteins [27], for evolutionary biology [28] and for a growing range of biomedical research areas outlined in this edition.

**Fig. 1:**
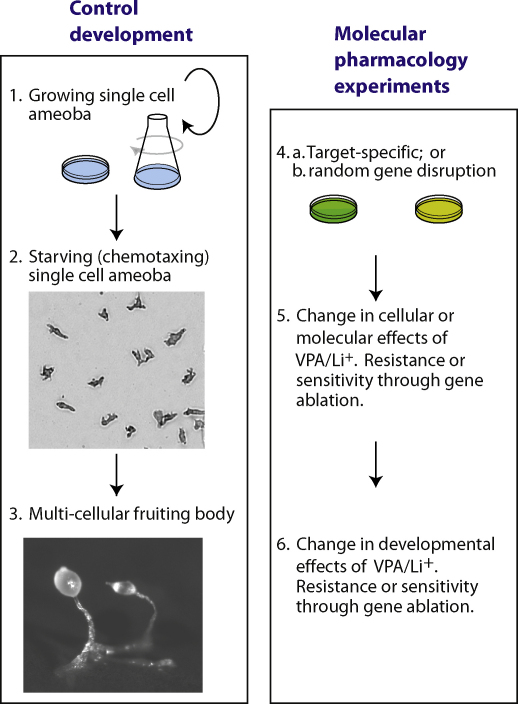
*Dictyostelium* as a simple model for molecular pharmacology research. Under control growth conditions; (1) *Dictyostelium* cells multiply by binary fission either in liquid media or in association with bacteria as a food source, until resources are depleted. (2) Induction of starvation causes cells to stop diving and enter a developmental phase, whereby expression of a range of developmental genes enables cells to move together in a process called chemotaxis (where cells move towards cyclic AMP). (3) Following aggregation of cells, *Dictyostelium* undergoes developmental differentiation to form a multi-cellular fruiting body, of around 1 mm in height, consisting primarily of spore and stalk cells. The development of molecular cell biology techniques for *Dictyostelium* research has enabled this model to be used in molecular pharmacology studies using either: (4a) Cell lines containing single or multiple ablated (or over-expressed) genes (green); or (4b) Pools of random insertional mutants (prepared using REMI; yellow) to provide mutant libraries for drug resistance screens. (5) The cellular role of pharmacological treatments (such as VPA and lithium (Li^+^)) can be analysed using either wild type cells, or cell lines containing ablated or over-expressed genes. (6) Analysis of resistance to the developmental block by VPA/Li^+^ can also help to understand and characterise the role of specific genes or identify novel genes involved in the action of bipolar disorder treatments. (For interpretation of the references to color in this figure legend, the reader is referred to the web version of the article.)

*Dictyostelium* can be cultured by either growth in nutrient rich media (ingesting media via macropinocytosis) or in association with bacteria as a food source (ingesting bacterial cells via endocytosis), and will continue to divide by binary fission until food or nutrients are depleted. Thus, in the presence of sufficient nutrients, large quantities of isogenic cells can be cultured and used for biochemical or cell signalling studies (Fig. 1). *Dictyostelium* also has an unusual part of its life cycle, whereby starvation initiates a development cycle and a range of genes are transcribed enabling chemically-directed cell movement (chemotaxis) and aggregation of ∼100 000 cells to a single point, followed by a primitive developmental process leading to the formation of distinct cell types (spore and stalk cells) in a mature fruiting body (Fig. 1). This development process takes around 24 h.

The haploid genome of this *Dictyostelium* has been fully sequenced [29] and contains 34 Mb of DNA which is 84-fold smaller than the human genome, but encodes 12,500 genes. Many of these genes now contain considerable annotation by the online research resources [Dictybase.org [30]]. Unusual aspects of the *Dictyostelium* genome include a high AT/GC ratio (86:14) and an average open reading frame of 1756 bp with 1.9 introns per gene. Thus, *Dictyostelium* provides a simple model system that is well-characterised at a genetic level.

The use of *Dictyostelium* as a pharmacological and pharmacogenetic molecular model for VPA and lithium was initially based upon the observation that both drugs block development, thus enabling a range of studies to understand their mechanism(s) of action (Fig. 1):

• REMI mutagenesis (Restriction Enzyme Mediated Insertional mutagenesis [31–33] provides an approach to create a library of insertional mutants, whereby a selectable marker is randomly integrated throughout the genome. The resulting pool of mutants can then be screened for resistance to VPA or lithium. Mutants with resistance to the block in development will identify the loci that control VPA or lithium action [34].
• Specific genes can be deleted within the haploid genome, and clonal cell lines can be isolated and used for biochemical or developmental resistance studies. The recent adaption of Cre-Lox based excision technology in *Dictyostelium* now enables the sequential repetition of this process, and thus the targeted ablation of multiple genes in a single cell line [35]. For example, Kay and co-workers recently ablated six genes in a single cell line [36].
• Over-expression of genes, or the expression of fluorescently tagged genes can be readily carried out [37], and isogenic cell lines can be isolated and used for studies examining the effect of VPA and lithium on cell signalling or development.

Thus *Dictyostelium's* unique biology allows a range of pharmacological studies to explore the role of VPA and lithium in regulating cell signalling and multi-cellular development.

## 4 A summary of valproic acid's molecular mechanisms of action

Valproic acid is a simple branched, short chain fatty acid (2-propylpentanoic acid), that was accidently discovered to function in seizure control in the early 1960s [38]. Since then its role has expanded to include its application as a mainstay treatment for bipolar disorder [39,40] and as a widely used prophylactic treatment for migraine [41]. More recently, it has also been proposed as a new treatment for a variety of conditions including ischemia [42], cancer [43], Alzheimer's disease [44], latent HIV [45], in terminal blood loss [46] and traumatic brain injury [47]. In addition to the beneficial effects of VPA, it also has a number of significant side effects. These include teratogenicity [48], whereby unborn children have a significantly increased chance of birth defects if the drug is taken by the mother during the first trimester of pregnancy [49], hepatotoxicity [50] and a range of common adverse side effects including weight gain, tremor, alopecia [51].

Not surprisingly, this gamut of therapeutic roles for VPA has been associated with a variety of cellular mechanisms. These range from increasing gamma aminobutaric (GABA) levels [41], reducing *de novo* inositol synthesis [52,53] and inositol trisphosphate depletion (InsP_3_) [54,59,99], to reducing ion channel activity [55], in regulation of fatty acid signalling and turnover [56] and in the activation of the mitogen activated protein (MAP) kinase pathway [57]. A role for VPA in GSK3 signalling has also been proposed, initially through direct inhibition [58], although this could not be reproduced by other groups [59,60]. Subsequent experiments showed contrasting results for an indirect effect of VPA on GSK3, primarily by elevated soluble β-catenin levels following treatment [61]. Histone deacetylase inhibition [60] has also been shown to provide a mechanism of action for VPA—an effect linked to teratogenicity [62].

## 5 A summary of lithium's molecular mechanisms of action

The mechanism of action for lithium has been of considerable interest since its re-discovery as a bipolar disorder treatment by Cade [63]. Lithium is the most commonly used treatment for bipolar disorder, and it is also effective in reducing pathological processes in a variety of Alzheimer's disease models [64]. Side effects include mild tremor, nausea, diarrhoea and its therapeutic window is narrow thus requiring regular monitoring of lithium blood concentration [65]. Furthermore, lithium has been proposed to be a teratogen since administration during pregnancy also increases the risk of congenital heart defects [66].

Despite the simple nature of this molecule (the third element on the periodic table), it still remains unclear how lithium functions in its therapeutic action. However, it has been widely shown to regulate four signalling pathways potentially associated with bipolar disorder: the inhibition of the enzyme glycogen synthase kinase (GSK3) [67,68], the activation of the MAPK signalling pathway [69], the attenuation of inositol signalling [54] and more recently phosphoinositol signalling [70].

A pharmacological approach to understand how VPA and lithium regulate cell signalling therefore requires the understanding of how these various pathways are modulated, and what genes and pathways are responsible for these drug effects. Here we will focus on the regulation of the MAP Kinase pathway and the inositol and phosphoinositol-dependent pathways.

## 6 MAP Kinase and bipolar disorder

Bipolar disorder has been associated with changes in neurotrophic signalling cascades, neuronal cellular atrophy and regional reductions in brain volume [71,72]. Structural imaging studies of patients with bipolar disorder have demonstrated reduced grey matter volumes in areas of the orbital and medial prefrontal cortex, ventral striatum and hippocampus and enlargement of third ventricles relative to healthy control patients [73,74]. This effect has also been shown in post mortem neuropathological studies with reductions in cortex volume [74], glial cell counts [75] and neuron size in various regions of the prefrontal cortex and amygdala of bipolar disorder patients [73]. Glial cell reductions in these regions are particularly interesting due to the role of these cells in regulating the development and maintenance of synaptic networks. Changes in neurotrophic signalling and atrophy may thus provide the mechanism of bipolar disorder pathology.

Lithium and VPA treatment have been shown to reverse or attenuate brain structural changes found in bipolar disorder [76,77], suggesting this neuroprotective effect to be the key mechanism of bipolar disorder treatment. The effect is thought to occur through the MAPK pathway by increasing the activation (phosphorylation) of the extracellular signal-regulated kinase 2 (ERK2). Increased phosphorylation of ERK2 (pERK2) in response to treatment is seen in cultured neuronal cells, rat hippocampus and frontal cortex and cultured rat thyroid cells [78–80]. As the MAPK pathway has been shown to modulate processes such as neuronal differentiation, neuronal survival and long term neuroplasticity it is likely to be involved in protecting against bipolar disorder-dependent neuronal death and is thus increasingly seen as the potential origin of the disorder [81]. Elucidating how VPA and lithium cause this effect is therefore of great therapeutic interest in understanding bipolar disorder aetiology [80,82].

**Fig. 2:**
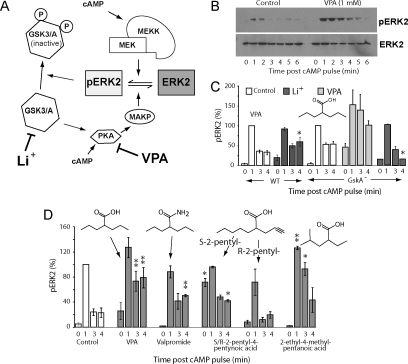
MAPK regulation by bipolar disorder treatments in the simple biomedical model *Dictyostelium*. In both *Dictyostelium* and mammalian systems, valproic acid (VPA) and lithium (Li^+^) treatment leads to an increase in activated MAPK, illustrated here for the phosphorylation of the ERK2 (extracellular regulated kinase 2 [ERK2] to form pERK2). (A) Regulation of pERK2 levels is complex, provided by a balance between kinase-dependent phosphorylation (catalysed by MEKK [mitogen-activated protein kinase kinase kinse], and MEK [MAPK kinase kinase]) and by de-phosphorylation (catalysed by MKP [MAP kinase phosphatase] in the PKA [protein kinase A] and GSK3/A [glycogen synthase kinase 3/A] signalling pathway). (B) Treatment of *Dictyostelium* cells with a single pulse of cyclic AMP (cAMP) gives rise to a transient increase in pERK2 detected by using phosphorylation-specific antibodies. Pre-incubation of *Dictyostelium* cells with VPA (1 mM, 60 min) increases and elongates the pathway activation by an unknown mechanism. (C) Research in *Dictyostelium* enables the use of isogenic cell lines containing either ablated or over-expressed genes, providing valuable tools for pharmacological research. For example, we can examine the role of GSKA (homologous to the human GSK3 proteins) in this effect by comparing pERK2 levels in wild-type cells and those lacking GSKA, under control conditions (no added drug) or following Li^+^ (10 mM, 60 min, dark grey) OR VPA (1 mM, 60 min, light grey) treatment. GSKA ablation increases pERK2 levels, suggesting that it functions as a negative regulator of this pathway, as does pharmacological inhibition with Li^+^. The lithium-dependent increase in pERK2 is also significantly reduced in the GSKA null, in agreement with a lithium/GSKA-dependent mechanism of action. In contrast, VPA causes a hypersensitive pERK2 increase in this mutant, suggesting VPA functions through a GSKA-independent mechanism. This approach shows an unambiguous role of GSKA in the transient formation of pERK2. * P < 0.05 compared to control. (D) Novel compounds related to VPA can also be analysed in structure–activity relationship studies (SARs), where for example, the amide derivative of VPA (valpromide), enantiomeric compounds (R- and S-2-pentyl-4-pentynoic acid), and altered branch derivatives (2-ethyl-4-methylpentanoic acid) show altered pathway activation. **P < 0.01, *P < 0.05 compared to control.

Employing *Dictyostelium* has shown that both VPA and lithium also up-regulate the activation of the MAPK pathway [82] by increasing pERK2 levels in this simple biomedical model system (Fig. 2A and B). In the case of VPA, this effect was shown to occur rapidly (within 10 min)—suggesting a biochemical basis for drug action rather than genetic regulation. The mechanism of action was suggested to be independent of upstream regulation, since it was not dependent on receptor phosphorylation (the cAMP receptor 1; cAR1) and to be independent of G protein dissociation (using FRET analysis or genetic deletion of G protein signalling). Furthermore increased pERK2 levels could not be phenocopied by reducing PIP_3_ production (using a widely-employed inhibitor to PI3K-family proteins, LY294002). However, a range of experiments suggested that VPA may function through blocking de-phosphorylation of pERK2 (Fig. 2). These experiments showed that pERK2 level upregulation is phenocopied by a reduced cAMP production (through ablation of adenylate cyclase A); pharmacological inhibition of protein kinase A (PKA) activity and pharmacological inhibition of phosphotyrosine phosphatase activity (by vanadate)—all implicating de-phosphorylation of pERK2 as the mechanism of action for VPA. The interaction of MAPK and cAMP/PKA pathway is complex [83] with multiple points of interaction and cross-regulation. Further analysis of this crossover in both *Dictyostelium* and mammalian systems will be necessary to better define the mechanism of VPA action on this pathway. The results also strongly implicate GSK3/A inhibition in reducing extended pERK2 levels, since both lithium and a specific GSK3/A inhibitor cause increased pERK2 levels at three and four minutes post stimulation [82], as does genetic ablation of GSKA in isogenic cell lines (Fig. 2C). This work also suggests that the lithium-induced pERK2 up-regulation is likely to be caused through direct inhibition of GSK3/A activity, leading to a decrease in PKA/MKP activity. This reasoning is also supported by the observation that ablation of GSK3/A blocks lithium-catalysed extended pathway activation at four minutes post stimulation (Fig. 2A,and C). These results suggest that VPA increases activated MAPK levels through inhibition of a cAMP/PKA dependent de-phosphorylation of pERK2 whilst the lithium-dependent effect occurs through GSKA/3-dependent signalling.

One important aspect of VPA pharmacology is the use of compounds related to VPA to distinguish between different targets in cell signalling. These studies were initially based upon the identification of reactive metabolites of VPA. For example β-oxidation of VPA gives rise to the metabolite E-2-propylpent-2-enoic acid whereas the action of the enzyme P450 results in the formation of 3-propylpent-4-enoic acid. These compounds show different inhibitory activity towards β-oxidation [84]. Furthermore, the teratogenic effect of VPA is also highly structurally dependent [62], requiring a carboxylic acid head group, a branch point on the second carbon, a hydrogen on the second carbon, and enantiomeric specificity (S-enantiomers show greater teratogenicity than R-enantiomers).

In *Dictyostelium* we have employed a range of VPA-related compounds to investigate the structural requirements for pERK2 up-regulation, and used this to differentiate this effect from other VPA sensitive pathways, mechanisms of action and therapeutic effects. For example, the amide derivative of VPA (valpromide) shows improved efficacy in seizure control [85] and lacks teratogenicity but shows only intermediate up-regulation of pERK2 levels in *Dictyostelium* (Fig. 2D). The elevation of pERK2 is also enantiomer specific since S-2-pentyl-4-pentynoic acid is strongly activating compared to its enantiomer R-2-pentyl-4-pentynoic acid that shows little effect (Fig. 2D). Interestingly, these two enantiomers have been shown to improve learning and memory with the more teratogenic S enantiomer exhibiting a stronger potency [86], however this increase was lost due to the detrimental effect on neuronal cell survival caused by teratogenicity. These results would support a role for pERK2 up-regulation in increased memory [86,87], as well as the possibility of increased protection against neuronal damage during bipolar disorder. The *Dictyostelium*-based studies also identified other, non-teratogenic VPA analogues (e.g. 2-methyl-2-pentenoic acid; Fig. 2D) that show equal efficacy to VPA, thus presenting potentially novel treatments for bipolar disorder. In support of a cAMP/PKA dependent mechanism of bipolar disorder drug action, lithium, carbamazepine [88,89] and VPA [90–92] inhibit cAMP-based signalling in mammalian systems.

Future work regarding VPA-catalysed inhibition of the cAMP/PKA/GSK3 signalling may provide mechanisms for the neuroprotective effects observed in treating bipolar disorder patients [57,80].

## 7 Inositol and phosphoinositol attenuation and bipolar disorder

Reduced inositol-dependent signalling was the first proposed mechanism for bipolar disorder treatments [54,93], primarily based on a lithium-induced attenuation of inositol recycling through inhibition of inositol monophophatase (IMPase) and inositol polyphosphate 1-phosphatase (IPPase) [94]. *Dictyostelium* has since proved an important model for understanding the role of VPA and lithium in regulating inositol signalling.

**Fig. 3:**
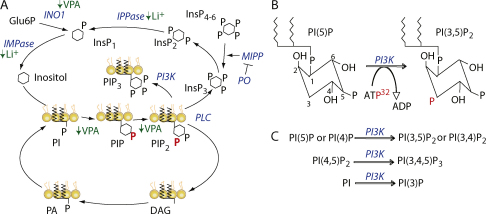
Inositol phosphate and phosphoinositide signalling regulation by bipolar disorder treatments in the simple biomedical model *Dictyostelium*. (A) Inositol signalling initiates from the phospholipase C (PLC)-generated cleavage of membrane-bound phosphatidylinositol 4,5-biphosphate (PIP_2_) to produce diacylglycerol (DAG) and cytosolic inositol 1,4,5-triphosphate (InsP_3_). Prolyl oligopeptidase (PO) negatively regulates multiple inositol polyphosphate phosphatase (MIPP) which in turn catalyses higher order inositol (InsP_4_, InsP_5_, InsP_6_) breakdown to form InsP_3_. InsP_3_ is hydrolysed to InsP_2_ and further broken down by Inositol polyphosphate phosphatase (IPPase) and inositol monophophatase (IMPase) to produce inositol. This is then incorporated into phosphoinositol signalling to form phosphatidylinositol (PI), phosphatidylinositol phosphate (PIP) and then PIP_2_. DAG is recycled via phosphatidic acid (PA). Lithium plays a well-documented role in inhibiting IMPase and IPPase to reduce inositol-based signalling, and VPA has been suggested to reduce *de novo* inositol biosynthesis through indirect inhibition of inositol synthase 1 (INO1), and the turnover of phosphoinositides (all indicated in green). (B) The inositol head group of the family of phosphoinositide compounds act as a substrate for a range of kinases, providing a dynamic and complex network of products and substrates. Addition of radio-labelled phosphate (red) to these kinase reactions gives rise to incorporation of radiolabel into defined places on the inositol ring (shown here for phosphatidylinositol 3-kinase (PI3K)). (C) Multiple substrates (shown here for PI3K) produce multiple products. Inositol ring (hexagon), P (black) phosphorylation site, P (Bold red) identified through *Dictyostelium* radio-labelling turnover experiments, enzymes in blue (italics); ↓ decreased. (For interpretation of the references to color in this figure legend, the reader is referred to the web version of the article.)

Our previous work identified a common effect of VPA and lithium in causing a reduction in InsP_3_ signalling in *Dictyostelium* [95] (Fig. 3). These studies identified prolyl oligopeptidase (PO), in a REMI mutant screen for resistance to the effect of lithium in blocking development [93,95] (Fig. 1). The PO knockout mutant was able to overcome this block and hence form fruiting bodies, by elevating InsP_3_ levels. These studies also showed that VPA, lithium and one other bipolar disorder treatment (carbamazepine) induced a common change in mammalian neurons, and that this change could be reversed by the addition of exogenous inositol or by inhibition of PO activity (Fig. 3) [59,93]. This loss of PO activity gave rise to an increase in the breakdown of InsP_4_, InsP_5_ and InsP_6_ to form InsP_3_. PO-dependent regulation of cellular InsP_3_ levels has since been demonstrated in astroglioma and COS-7 kidney cell lines [96,97]. Thus, PO ablation reverses the common effect of bipolar disorder drugs. This was a novel discovery, since the role of PO was thought to be entirely based upon the cleavage of small peptides containing prolyl residues [98]. A link between PO enzyme activity and bipolar disorder has also been reported in patients that exhibit increased enzyme activity [21]. Furthermore, both *Dictyostelium* and mammalian systems have been used to identify a range of compounds structurally related to VPA that cause inositol depletion to show that this effect is not correlated with teratogenic activity [59,99].

The mechanism of lithium-dependent inositol phosphate regulation has recently been further characterised in *Dictyostelium* [100] (Fig. 3). King et al. [100] showed that chemotaxis provides a suitable model for examining the effects of lithium in *Dictyostelium,* since it causes a strong reduction in cell speed. Ablation of PO activity reduced sensitivity to this lithium effect, consistent with an InsP_3_-dependent mechanism of lithium action. Ablation of the enzyme multiple inositol polyphosphate phosphatase (MIPP), responsible for breakdown of InsP_4–6_, resulted in reduced InsP_3_ levels, whereas MIPP over-expressing cell extracts had an enhanced activity compared to wild type controls. MIPP activity was necessary for PO-inhibition dependent inositol phosphate regulation both in chemotaxis and in the production of InsP_3_. These data clearly establish PO as the regulator of MIPP activity. However, surprisingly, over-expression of MIPP gave a lithium hyper-sensitive phenotype.

The origin of lithium hyper-sensitivity in cells over-expressing MIPP was found to be due to the elevated expression of a range of genes related to inositol signalling [100]. These included IMPase and IPPase—previously been described as cellular targets of lithium [94], and inositol synthase 1 (INO1)—responsible for the *de novo* synthesis of inositol. The increase in gene expression was also observed following both genetic and pharmacological inhibition of PO activity. However in all cases, this increased expression was dependent upon the presence of MIPP activity and was also induced by over-expression of the kinases responsible for the production of InsP_4–6_ from InsP_3_. Thus this work identified a gene regulatory mechanism where the activation or over-expression of MIPP leads to elevated expression of a range of inositol regulation genes in *Dictyostelium*. The results were then successfully translated to human cells (HEK293) to demonstrate that PO inhibition also up-regulates several IMPase genes and that this elevation is dependent on MIPP activity. It is of interest that IMPase polymorphisms have been associated with risk of bipolar disorder in family studies [101–103].

A new mechanism of action for bipolar disorder drugs has been recently proposed based upon work in *Dictyostelium* [104,105]. Initially, to characterise a VPA-dependent block in *Dictyostelium* cell movement, we investigated the role of phosphoinositide signalling as a target for VPA action [104]. VPA treatment produced a rapid and strong reduction of phosphoinositide production. Further investigation of this effect in *Dictyostelium* employed cells transformed with a PIP_3_ binding protein (PH_crac_-GFP) enabling the time- and location-dependent monitoring of PIP_3_ production. VPA produced an acute (10 min) reduction in PIP_3_ production, also shown by reduced PIP_2_ and PIP levels using *in vivo* radio-labelling experiments (Fig. 3A and B, red). These effects were phenocopied by pharmacological inhibition of phosphatidylinositol 3-kinase (PI3K) using the inhibitor LY294002. Identification of the mechanism that cause the inhibitory activity on phosphoinositide signalling is highly complex, since phosphorylation of the phosphatidylinositol head group can occur at three positions on the inositol ring (Fig. 3), creating a variety of products including single phosphorylated moieties (e.g. PI(3)P), as well as double and triple phosphorylated products (e.g. PI(3,4)P_2_ and PI(3,4,5)P_3_). This data, for the first time, implicated phosphoinositide signalling as a potential target for bipolar disorder treatments. Considerable evidence suggests that elevated phosphoinositol signalling may be an underlying cause of bipolar disorder since a variety of studies have identified this effect in bipolar disorder drug-free patients [106–108] as well as the subsequent down-regulation of phosphoinositide levels following bipolar disorder drug treatment [108,109].

Recent work on the effects of lithium on *Dictyostelium* has now extended the observation that bipolar disorder-treatment attenuates phosphoinositide-mediated signalling [70]. The analysis of the mechanism leading to decreased cell speed following treatment indicated this effect is independently of both cAMP synthesis and F-actin polymerisation. However, the speed reduction was phenocopied by pharmacological or genetic ablation of PI3K activity using either LY294002 or a single *Dictyostelium* cell line lacking all five PI3K genes [36] implicating phosphoinositide signalling as a target for lithium treatment. In agreement with this, the phosphorylation of the protein kinase B (PkbA) homologue, whose membrane translocation is dependent on binding to the PH domain of PIP_3_, was also decreased following lithium treatment compared to untreated cells. Furthermore, these effects also occurred in conjunction with a decrease in the rate of PIP and PIP_2_ synthesis, although no changes in steady-state concentration of PIP_2_ levels were detected. This may be due to a small rapidly turned over pool of PIP_2_ that is quickly metabolised during chemostaxis providing a lithium sensitive target. Since the mechanism for inositol signalling attenuation by lithium is known to function through the uncompetitive inhibition of IMPase and IPPase [94], King et al. [70] over-expressed IMPase to reverse the effects of lithium in PIP/PIP_2_ synthesis and PkbA phosphorylation. The results were then successfully reproduced in a human neutrophil cell line (HL60) where the translocation of a PIP_3_-binding GFP construct to cell membranes (as a readout for PIP_3_ production) was reduced following lithium treatment. Based on these studies in *Dictyostelium* investigating both VPA and lithium mechanisms of action, we can now propose a new extension of the current of inositol depletion hypothesis, whereby bipolar disorder treatments give rise to attenuated phosphoinositide signalling.

## 8 Conclusion/summary

To help those diagnosed with bipolar disorder, ongoing research endeavours to identify the cell signalling changes that are targeted by the pharmacological agents, valproic acid and lithium. This approach overcomes the multitude of potential loci implicated in the aetiology of the disorder, but instead focuses on understanding the mechanism(s) leading to symptomatic control. It also enables the identification of new compounds that may be more efficacious or have reduced side effects in bipolar disorder treatment. Research in this area has traditionally used rodents as model organisms, however the increasing use of other simple model systems such as *Dictyostelium* provides distinct advantages.

Investigation of cell signalling regulated by VPA and lithium demonstrate that both *Dictyostelium* and the animal brain respond by elevating pERK2 levels in the MAPK pathway. Analysis of this effect in *Dictyostelium* has proposed two mechanisms: VPA regulates increased pERK2 levels through inhibition of cAMP/PKA signalling, whereas lithium inhibits GSK3/A, with both effects leading to the reduced dephosphorylation of pERK2. The strong structural specificity of this effect in regards to VPA suggests novel compounds may be identified that show improved efficacy for MAPK regulation.

Another cell signalling effect of VPA and lithium in *Dictyostelium* is the reduction of inositol-dependent signalling. Recently studies have suggested that cellular inositol phosphate levels are controlled by both inositol recycling and the MIPP-catalysed breakdown of higher-order inositol phosphate compounds (InsP_4–6_) regulated by prolyl oligopeptidase activity. This work has also discovered a transcriptional-based effect, whereby lithium treatment gives rise to altered transcriptional regulation of inositol-signalling related genes. In addition *Dictyostelium*-based research has discovered a potential key pharmacological target of VPA and lithium in attenuating phosphoinositol signalling, with both drugs causing an acute attenuation of phosphoinositol turnover. In the case of lithium, it is likely that this effect is through inositol signalling attenuation, whereas the mechanism controlled by VPA remains to be determined. These discoveries open up a exciting field of phosphoinositol research, with the promise of new therapies (based around the structure of VPA) showing increased efficacy in phosphoinositide regulation.

This review has summarised current research aimed at unravelling the complex mechanisms of VPA and lithium in regulating cell signalling, and the important role of *Dictyostelium* as a simple biomedical model for this research. A better understanding of these cell signalling changes may ultimately identify the underlying causes of bipolar disorder and will enable the development of better therapeutic treatments.
